# Light chain deposition disease masquerading as hypophosphatemic osteomalacia: a case report

**DOI:** 10.3389/fmed.2026.1849258

**Published:** 2026-05-21

**Authors:** Jiannan Chen, Qi Bing, Maodong Liu, Song Zhao, Qian Wang, Qi Hu, Zhe Zhao

**Affiliations:** 1 Department of Neurology, Hebei Medical University Third Hospital, Shijiazhuang, Hebei, China; 2 Department of Nephrology, Hebei Medical University Third Hospital, Shijiazhuang, Hebei, China

**Keywords:** Fanconi syndrome, hypophosphatemic osteomalacia, kappa light chains, light chain deposition disease, renal biopsy

## Abstract

Light chain deposition disease (LCDD) is characterized by the deposition of monotypic immunoglobulin light chains in multiple organs. The kidneys are usually affected in cases of LCCD, and they typically present with mesangial nodule formation, extensive monoclonal light chain deposition, proteinuria, and renal insufficiency. We report a rare case of LCDD that presented with bone pain and proximal amyosthenia secondary to renal tubular phosphate loss. A 42-year-old woman presented with progressive pain in the waist and bilateral hips, along with increasing amyosthenia in both lower limbs that had persisted for more than a year. Upon investigation, she was diagnosed with hypophosphatemic osteomalacia (HO). She also had aminoaciduria, renal glucosuria, hypokalemia, hypouricemia, and renal tubular proteinuria with elevated serum kappa light chains. A renal biopsy revealed the presence of fibrillar deposits of kappa light chains along the glomerular basement membranes, as observed using electron microscopy and immunofluorescence. Congo red staining was negative. Based on the ‌finding of abnormal plasma cells comprising 1.1%‌ of cellular elements in a bone marrow biopsy, we made a diagnosis of monoclonal gammopathy of renal significance. The patient received chemotherapy and exhibited positive clinical and biochemical responses. This case describes a unique presentation of renal injury due to LCDD masquerading as HO. The aim of this report is to highlight that HO in adults is usually acquired and that addressing the underlying etiology can result in a successful cure.

## Introduction

Light chain deposition disease (LCDD) is associated with underlying plasma cell dyscrasias or other lymphoproliferative disorders and is characterized by the deposition of monotypic immunoglobulin light chains in multiple organs (1). Light chains are filtered by the glomeruli, reabsorbed in proximal tubules via receptor-mediated endocytosis, and degraded within tubular cells by lysosomal enzymes; consequently, the kidneys are usually affected (2, 3). LCDD typically presents with mesangial nodule formation, extensive monoclonal light chain deposition, proteinuria, and renal insufficiency (4). Additionally, hepatic, cardiac, and neural deposits have been documented. Hypophosphatemic osteomalacia (HO) as the initial manifestation of LCDD has not been previously reported. We report a rare case of LCDD that presents with bone pain and proximal amyosthenia.

## Case presentation

A 42-year-old woman presented with progressive pain in the waist and bilateral hips, along with increasing amyosthenia in both lower limbs that had persisted for more than a year. Coughing, walking, and bending over aggravated the pain. The physical examination was unremarkable. Laboratory investigations revealed normal levels of hemoglobin, white blood count, platelet count, erythrocyte sedimentation rate, serum albumin and globulin, albumin/globulin ratio, serum sodium, serum calcium, serum glucose, parathyroid hormone (PTH), and 25-HydroxyvitaminD. However, several indicators were abnormal: serum creatinine at 87.25 umol/L (normal 41–73 umol/L), estimated glomerular filtration rate (eGFR) at 65.6 mL/min, serum urea at 1.84 mmol/L (normal 2.6–7.5 mmol/L), serum potassium at 3.15 mmol/L (normal 3.5–5.3 mmol/L), serum chloride at 111.5 mmol/L (normal 99–110 mmol/L), serum phosphate at 0.66 mmol/L (normal 0.85–1.51 mmol/L), serum alkaline phosphatase at (ALP) 189 U/L (normal 35–100 U/L), and serum uric acid at 68 mmol/L (normal 155–357 mmol/L). The urine analysis revealed protein level of 1+, occult blood at 3+, and urine glucose at 4+. The 24 h urine protein excretion was 3.14 g (normal 0.04–0.23 g). Further analysis of the urine protein showed that 48.5% was of renal tubular origin, 20.5% was of glomerular origin, and 31% was of albumin. Specifically, immunoglobulin levels were found to be 25.44 ug/ml (normal 0–6 ug/ml), while microalbumin levels were >50 ug/ml (normal 0–10 ug/ml). Additionally, urine Bence Jones protein was measured at 10ug/ml (normal 0–0.195 ug/ml). Urine tests for congenital metabolic defects indicated glucosuria, lactaturia with ketonuria, and amino aciduria. Serum and urine electrophoresis demonstrated positive kappa free light chains, with normal serum IgA, IgG, IgM, and lambda free light chain levels. Analysis of serum-free light chains revealed kappa and lambda levels of 1,085 mg/L and 15.2 mg/L, respectively. ^99^mTc-MDP bone scintigraphy revealed multiple foci of increased tracer accumulation in the bilateral ribs, left sacroiliac joint, right proximal femur, and both feet (Figure 1), while radiographs and MRI of the pelvic bones did not show osteolytic lesions. Dual-energy x-ray absorptiometry demonstrated low bone mineral density (L1-4: T-score −2.5, Z-score −2.2; Left femoral neck: T-score −2.2, Z-score −2.0; Right femoral neck: T-score −3.5, Z-score −3.0).

**Figure 1:**
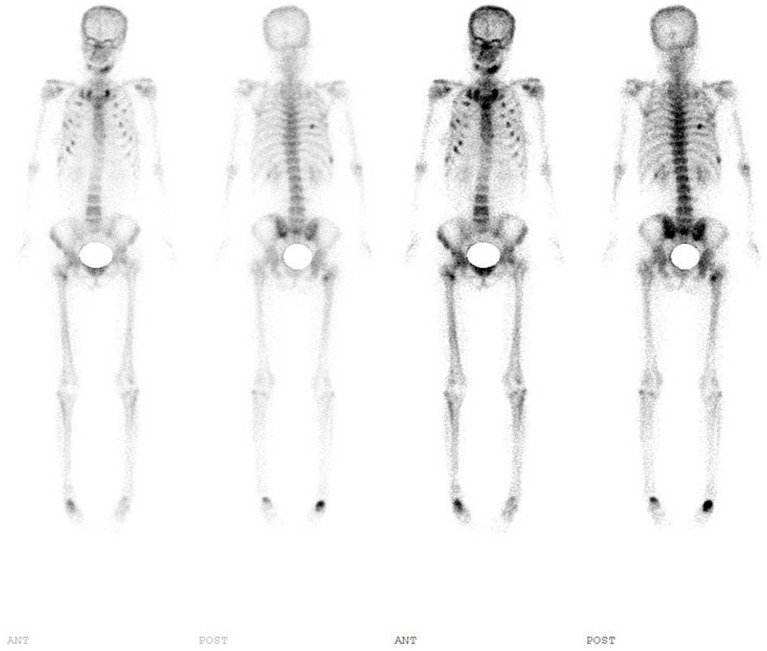
^99m^Tc-MDP bone scintigraphy revealed multiple foci of increased tracer accumulation in the bilateral ribs, left sacroiliac joint, right proximal femur, and both feet.

A renal biopsy was performed in view of nephrotic damage. Light microscopy of 33 sampled glomeruli revealed 2 glomeruli with ischemic sclerosis. The remaining glomeruli exhibited mild mesangial cell and matrix hyperplasia. ‌Non-argyrophilic amorphous protein deposits‌ were observed in the mesangium. ‌Glomerular basement membranes (GBMs) appeared diffusely thickened (mild-to-moderate) and were ribbon-like. Significant granular and vacuolar degeneration was noted in renal tubular epithelial cells, accompanied by brush border attenuation, luminal dilation, and focal tubular atrophy (approximately 5%). ‌Focal interstitial fibrosis‌ (approximately 5%) with mononuclear cell infiltration and ‌arteriolar wall thickening‌ was identified. Congo red staining was negative. Immunofluorescence demonstrated ‌linear staining (weakly positive) for kappa and lambda light chains, IgG, and IgA along glomerular capillary walls‌. IgM, C3, C1q, fibrinogen-related antigen (FRA), and albumin were negative. Light chain deposits along tubular basement membranes (TBMs) or arteriolar myocytes were not observed. Electron microscopy‌ (EM, Department of Pathology, Peking University Health Science Center) revealed ‌diffuse thickening of GBMs‌. ‌Fibrillar deposits‌ measuring <10 nm in diameter were ‌arranged randomly‌. ‌Podocyte foot processes‌ exhibited extensive fusion. Renal tubular epithelium showed ‌increased lysosomes‌ and detachment of microvilli. ‌Immunofluorescence with antigen retrieval showed linear kappa light chain deposits along GBMs ‌(Figure 2). Bone marrow biopsy revealed ‌abnormal plasma cells comprising approximately 1.1%‌ of cellular elements. These cells exhibited ‌immunophenotypic expression‌ of CD38, CD138, CD56, CD27, and cytoplasmic kappa (cKappa).

**Figure 2:**
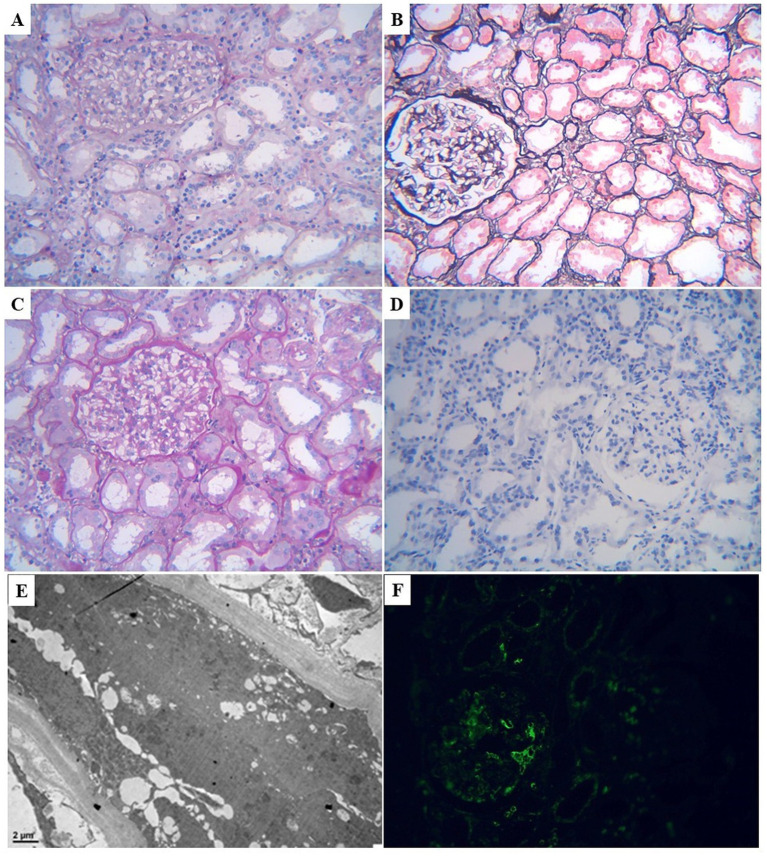
**(A)** Significant granular and vacuolar degeneration in renal tubular epithelial cells, accompanied by brush border attenuation, luminal dilation, and focal tubular atrophy (HE x200). **(B,C)** Mild mesangial cell and matrix hyperplasia. Non-argyrophilic amorphous protein deposits in the mesangium (B.PASM x200, C.PAS x200). **(D)** Negative Congo red staining (x200). **(E)** Increased lysosomes and detachment of microvilli are observed in renal tubular epithelial cells, without fibrillar or crystalline deposits (EM x10000). **(F)** Linear kappa light chain deposits along GBMs (immunofluorescence with antigen retrieval).

A final diagnosis of ‌monoclonal gammopathy of renal significance (MGRS) due to LCDD causing HO‌ was established. He was referred to the hematology department and was managed with periodic treatment of BCD (bortezomib, cyclophosphamide, and dexamethasone), neutral phosphate solution, and calcium supplements. There was a good clinical and biochemical response. Urine analysis showed negative protein and 1 + urine glucose. Serum electrophoresis revealed no monoclonal band, and serum phosphate levels were normal.

## Discussion

The patient presented with bone pains. Laboratory investigations indicated hypophosphatemia, elevated serum ALP, and reduced tubular maximum of phosphate reabsorption for glomerular filtration rate (TmP/GFR), alongside normal 25-hydroxyvitamin D levels. ^99m^Tc-MDP bone scintigraphy findings were consistent with osteomalacia. These features supported the diagnosis of HO. The concurrent presence of aminoaciduria, renal glucosuria, hypokalemia, hypouricemia, and renal tubular proteinuria established the diagnosis of Fanconi syndrome (FS). Evidenced by the elevated serum kappa light chains,‌ linear kappa light chain deposits along the GBMs with negative Congo red staining in renal biopsy, and 1.1% aberrant plasma cells in bone marrow biopsy, the LCDD diagnosis was confirmed.

HO is mostly caused by increased phosphorus excretion by the kidneys and is mainly seen in genetic diseases. A few causes are acquired, such as tumor-induced osteomalacia, FS, and drug-induced ones, which are more common in adults with slowly progressive symptoms of bone pains, muscle weakness, and recurrent fractures (5, 6). HO associated with FS‌ manifests not only with features of osteomalacia but also with ‌a broader spectrum of metabolic abnormalities‌. Due to generalized proximal renal tubular dysfunction, FS is characterized by low-molecular-weight proteinuria, aminoaciduria, hypophosphatemia, hypouricemia, renal glycosuria, and proximal renal tubular acidosis (7). A rare etiological factor for acquired FS is monoclonal gammopathy‌, encompassing monoclonal gammopathy of undetermined significance (MGUS), multiple myeloma, Waldenström macroglobulinemia, lymphoplasmacytic lymphoma, chronic lymphocytic leukemia, and monoclonal immunoglobulin deposition disease (MIDD) (8, 9). MIDD is a rare nephropathy characterized by the deposition of non-fibrillar, Congo red-negative, monoclonal immunoglobulin molecules in glomerular and tubular basement membranes (10). LCDD is the most common subtype, wherein deposits consist ‌exclusively‌ of monoclonal light chains and ‌nearly 91%‌ are of the kappa subtype (11). Approximately 10 to 30% of MIDD cases are secondary to malignant plasma cell diseases, such as multiple myeloma. MGRS refers to conditions that ‌do not fulfill‌ the diagnostic criteria for malignant neoplasms (abnormal plasma cells comprising < 10%, serum monoclonal immunoglobulin < 3 g/dL) (12).

Clinically, renal involvement in LCDD is pathologically typically characterized by nodular glomerulosclerosis, with linear light-chain deposition observed in GBMs, Bowman’s capsule, and around arteriolar myocytes in most cases (13). It is commonly held that patients with LCDD present with proteinuria, nephrotic syndrome, and elevated serum creatinine at diagnosis, while FS is uncommon (8). Isolated tubular deposition is even rarer and may involve any segment of the tubule (14). Previously, Tu et al. (8) reported monoclonal kappa light chain deposition solely along TBMs, with GBMs remaining negative. Furthermore, this patient’s clinical manifestations align with those in our case. Theoretically, the physicochemical properties and molecular size of monoclonal immunoglobulins determine the renal lesions they cause. Larger monoclonal immunoglobulin molecules containing both heavy and light chains cannot readily traverse the glomerular filtration barrier, leading to glomerular deposition and subsequent inflammation (proliferative glomerulonephritis or immunotactoid glomerulopathy). Conversely, low-molecular-weight light chains penetrate the filtration barrier to induce primarily tubular pathologies such as cast nephropathy (distal tubules) and light-chain proximal tubulopathy (LCPT) (proximal tubules). Finally, light or heavy chains complexed with other proteins may concurrently affect both glomerular and tubular structures, exemplified by monoclonal immunoglobulin-amyloidosis (AL and heavy-chain amyloidosis) and MIDDs (15). Thus, renal pathological changes in LCDD characteristically affect both glomeruli and renal tubules. On light microscopy, a characteristic histological finding is nodular sclerosing glomerulopathy‌, ‌observed in approximately 50 to 60% of cases.‌ Thickening and wrinkling of the TBMs, accompanied by ‌renal tubular atrophy and interstitial fibrosis, are present in virtually all patients‌. Vascular involvement occurs in 60 to 70% of patients, characterized by deposition within vascular myocytes‌. Immunofluorescence and EM possess pivotal diagnostic significance‌. Their hallmark feature is the linear deposition of light chains‌ along the GBMs, TBMs, and vascular walls (16). Li et al. (4) found that TBM deposits were revealed in 100% of patients, GBMs deposits in 68.8%, and vascular deposits in 52.1% on immunofluorescence. On EM, deposits were seen along the TBMs in 100% of patients and along the GBMs in 93.3% of patients. In Nasr SH’s report (11), all patients showed diffuse linear monoclonal deposits along GBMs and TBMs on immunofluorescence, and the deposits were seen along TBMs in 100% and along the GBMs in 95% of patients on EM.

However, in our case, clinical manifestations and urinalysis demonstrated predominantly tubular damage with mild glomerular involvement. The detection of deposits within the GBMs provided a pathological explanation for glomerular damage manifestations. Although renal tubular pathological changes could explain the patient’s FS manifestations, the absence of TBM deposits was inconsistent with typical LCDD findings. Factually, similar cases have been reported (17, 18). Additionally, Malhotra et al. (6) reported a unique case of renal AL amyloidosis mimicking FS-induced HO, featuring amyloid fibril deposition in the glomerular mesangium without corresponding TBM deposits on EM. The precise mechanisms underlying heterogeneous localization patterns remain largely unknown. Among MGRS-associated lesions, the crystalline variant of LCPT most commonly causes light-chain FS and is typically associated with kappa light chain deposition (19, 20). Geometric crystalline inclusions are characteristically observed in proximal tubular epithelial cells, whereas no such crystals were identified in our pathological examination. In LCDD, how do light chains impair the function of proximal renal tubules and lead to FS? Light chains are freely filtered through the capillary walls and delivered to the proximal tubules (21, 22). They are endocytosed in the proximal tubules through the megalin-cubilin receptor, and transferred to endosomes and lysosomes for degradation (23, 24). Failure of the endosomal system to catabolize abnormal light chains initiates pathological alterations (25). Batuman et al. (26) have also demonstrated the direct toxicity of light chains on proximal tubular cells, inhibiting the transport of phosphate and glucose into these cells and activating redox-sensitive pathways that promote apoptosis. Once the lysosomes burst and release their hydrolytic enzymes, cytoplasmic vacuolization, desquamation, and/or frank necrosis occur in proximal tubular cells. The light chains also accumulate in close relationship with the TBMs through transcytotic vesicular pathways (27), leading to the recruitment of inflammatory cells and interstitial inflammatory reactions (20).

Notably, another feature differing from classic granular deposits was the fibrillar deposits observed on EM in this case. Consequently, repeated Congo red staining following the EM report remained negative. Amyloidosis could therefore be reasonably excluded. Fibrillar deposits with negative Congo red staining in LCDD have also been documented (2, 18). Next, differentiation from other diseases exhibiting Congo red-negative fibrillar deposits is required, namely immunotactoid glomerulopathy (ITG) and fibrillary glomerulonephritis (FGN). ITG is a rare glomerulopathy and typically presents with renal insufficiency and nephrotic syndrome (28). Pathological features of ITG include electron microscopic identification of deposits consisting of parallel microtubules with hollow cores (>30 nm in diameter), light microscopic demonstration of a membranoproliferative glomerulonephritis pattern, and immunofluorescence detection of IgG, C3, and C1q within mesangial and capillary walls (29). FGN mainly presents with nephrotic syndrome, hematuria, and reduced glomerular filtration rate. Pathological features include mesangial and endocapillary proliferation on light microscopy, ‌polyclonal IgG and C3 deposition on immunofluorescence, and randomly arranged fibrils (12–22 nm) in the mesangium and GBMs on EM (30). Differently, the fibrillar deposits of the LCDD case consisted of kappa light chains, and the diameters were smaller than those in the two disorders. Additionally, the two diseases were mainly characterized by glomerular involvement, while the patient showed significant pathological and clinical manifestations of renal tubular damage.

This study has some limitations, notably that fibroblast growth factor 23 (FGF23) was not measured. FGF23 is now recognized as the principal regulator of phosphate homeostasis, acting on the proximal renal tubule to reduce tubular phosphate reabsorption. In addition, FGF23 inhibits expression of 25-hydroxyvitamin D3 1-alpha-hydroxylase in the proximal tubules, leading to inadequate levels of 1,25(OH)2D and subsequent decreased intestinal phosphate and calcium absorption (31). Thus, the net effects of excess FGF23 are hypophosphatemia, rickets, osteomalacia, and, frequently, secondary hyperparathyroidism (32, 33). In recent years, the differential diagnosis of HO has been based on FGF23 levels, classifying the condition as FGF23-dependent or FGF23-independent forms. This classification is critically important for identifying renal phosphate wasting caused by primary tubular injury and for determining the underlying etiology and appropriate treatment of osteomalacia. This is particularly relevant for tumor-induced osteomalacia, which is a major cause of acquired osteomalacia in adults (34, 35).

In summary, the patient’s clinical presentation was dominated by HO induced by FS, and the renal biopsy revealed Congo red-negative fibrillary deposits in the GBMs, which differed from the classic features of LCDD and posed diagnostic challenges. Adult-onset HO should be thoroughly investigated for an acquired cause. LCDD should also be considered even when renal tubular injury is the dominant manifestation.

## Data Availability

The original contributions presented in the study are included in the article/supplementary material, further inquiries can be directed to the corresponding author.
